# Letter to the editor: Antimicrobial resistance data and treatment guidelines – challenges in the context of urine samples and mecillinam testing

**DOI:** 10.2807/1560-7917.ES.2023.28.32.2300418

**Published:** 2023-08-10

**Authors:** Muna Abu Sin, Esther Wohlfarth, Anja Klingeberg, Tim Eckmanns, Michael Kresken, Evelyn Kramme, Guido Werner

**Affiliations:** 1 Unit Healthcare-associated Infections, Surveillance of Antimicrobial Resistance and Consumption, Department of Infectious Disease Epidemiology, Robert Koch Institute, Germany; 2 Antiinfectives Intelligence GmbH, Cologne, Germany; 3 Paul Ehrlich Society for Infection Therapy, c/o Antiinfectives Intelligence GmbH, Cologne, Germany; 4 Department of Infectious Diseases and Microbiology, University of Lübeck and University Hospital Schleswig-Holstein, Campus Lübeck, Lübeck, Germany; 5 Unit Nosocomial Pathogens and Antibiotic Resistances, Department of Infectious Diseases, Robert Koch Institute, Germany

**To the Editor:** In their important paper on antimicrobial resistance of Enterobacterales from routine diagnostic urine samples, Stoltidis-Claus et al. reported mecillinam resistance percentages of 17.9% among *E. coli* isolates from the catchment area of their laboratory in Germany over the study period from 2016 to 2021 [1]. Despite increasing pivmecillinam consumption in Germany, the authors detected a declining trend in resistance over this period. They discussed their findings in the context of other publications, which showed considerably lower percentages of resistance against mecillinam, and addressed potential unreliable results by automated testing.

The 2017 German Association of the Scientific Medical Societies (AWMF) guideline on uncomplicated community-acquired urinary tract infections, which is currently under revision, recommended pivmecillinam as first-line treatment among other antibiotics. Considering the high resistance against mecillinam reported by Stoltidis-Claus et al., one might question the use of pivmecillinam as first-line treatment.

As antibiotic treatment for uncomplicated community-acquired urinary tract infections is empirical, the availability of antimicrobial resistance data is important. However, routine diagnostic testing for uncomplicated urinary tract infection is not recommended and surveillance data as presented in this study tend to overestimate the resistance situation, as has been shown by Klingeberg et al. [2]. Therefore, surveillance data need to be interpreted with caution in order to inform treatment guidelines and limitations of those data need to be addressed transparently.

In a recent study by Kresken et al. published in 2022, 5.2% of tested *E. coli* isolates from urine specimens of primary care patients in Germany were resistant against mecillinam [3]. Notably, in this study, susceptibility testing was performed by agar dilution, which is considered as the gold standard. According to International Organization for Standardization (ISO) guideline 20776–2 [4], derived antimicrobial susceptibility testing (AST) methods should confirm comparability of their results.

The antibiotic resistance surveillance system in Germany (ARS) is a laboratory-based sentinel surveillance system that collects routine diagnostic data on pathogen identification and AST from in- and outpatients. An interactive publicly accessible database with selected drug-bug combinations is updated once a year [5]. The upcoming update in the third quarter of 2023 will include data on mecillinam resistance in *E. coli*. Preliminary ARS data show a decreasing trend in mecillinam resistance for *E. coli* isolates in urine samples from 7.9% (n = 196,028 tested isolates) in 2019 to 4.8% (n = 333,301) in 2022. In urine samples from outpatients, the percentages of resistance (2020: 5.9% (n = 141,724); 2021: 5% (n = 176,604)) are in line with those reported by Kresken et al. Regional stratification for outpatient samples from North Rhine-Westphalia for 2020 and 2021 showed mecillinam resistance percentages of 7.5% (n = 28,138) and 5% (n = 43,511) respectively. Furthermore, ARS data have the same limitations as the data by Stoltidis-Claus et al. in respect to sampling and automated testing.

A recent study by Klingeberg et al. showed some regional differences in mecillinam resistance of *E. coli* isolates from uncomplicated urinary tract infections with the highest percentages of 13% (95% confidence interval (CI): 4.5–32.1) for non-recurrent and 7.5% (95% CI: 1.3–31.5) for recurrent episodes in North Rhine-Westphalia – the main catchment area of the Stoltidis-Claus et al. study. The overall mecillinam resistance in this study was 4.3% (95% CI: 2.7–6.6) for non-recurrent and 5.1% (95% CI: 3.1–8.2) for recurrent uncomplicated urinary tract infections [6].

As the reported resistance data by Stoltidis-Claus et al. might impact antibiotic treatment decisions regarding first-line use of pivmecillinam and we could not explain the observed differences satisfactorily, we contacted the authors to discuss their findings. We agreed to further analyse isolates from the Kresken et al. study and from a more recent, yet still unpublished study by systematically comparing susceptibility results from different laboratory methods including agar dilution and different automated testing systems, which might bring some further insight to these contradictory data.
